# The Role of Dendritic Cells in Tissue-Specific Autoimmunity

**DOI:** 10.1155/2014/857143

**Published:** 2014-04-30

**Authors:** Jacques Mbongue, Dequina Nicholas, Anthony Firek, William Langridge

**Affiliations:** ^1^Center for Health Disparities and Molecular Medicine, 11085 Campus Street, Mortensen Hall, Department of Basic Sciences, Loma Linda University School of Medicine, Loma Linda, CA 92354, USA; ^2^Endocrinology Section, JL Pettis Memorial VA Medical Center, Loma Linda, CA 92354, USA

## Abstract

In this review, we explore the role of dendritic cell subsets in the development of tissue-specific autoimmune diseases. From the increasing list of dendritic cell subclasses, it is becoming clear that we are only at the beginning of understanding the role of these antigen presenting cells in mediating autoimmunity. Emerging research areas for the study of dendritic cell involvement in the onset and inhibition of tissue-specific autoimmunity are presented. Further, we compare tissue specific to systemic autoimmunity to demonstrate how development of dendritic cell-based therapies may be broadly applicable to both classes of autoimmunity. Continued development of these research areas will lead us closer to clinical assessment of novel immunosuppressive therapy for the reversal and prevention of tissue-specific autoimmunity. Through description of dendritic cell functions in the modulation of tissue-specific autoimmunity, we hope to stimulate a greater appreciation and understanding of the role dendritic cells play in the development and treatment of autoimmunity.

## 1. Introduction

Tissue-specific autoimmunity may be defined as a progressive inflammatory immune response to specific proteins originating from cells in a tissue or organ resulting in diminished organ function or organ failure. Organ destruction is currently thought to arise from dysregulation of the immune system. The identification of immune cells responsible for initiation of autoimmunity remained elusive until a new class of immune cells, dendritic cells (DC), was discovered in the early 1970s by Nobel Laureate Ralph Steinman. Until this time, cells representing the innate and adaptive arms of the immune system were considered to be separate entities. However, Steinman's discovery that dendritic cells were the “missing link” responsible for coordinating innate and adaptive immune responses has revolutionized the relationship between these two segments of the immune system. In addition, this discovery allowed the proliferation of many novel therapeutic strategies for prevention and treatment of tissue-specific autoimmunity. The first clues to interactions between innate immunity and the adaptive immune response became apparent in 1973 when Steinman and Cohn detected the presence of an unusual immune cell type while observing cells from mouse spleen by phase contrast microscopy [1, 2]. The authors identified a specific population of immune cells that did not display the typical morphology of macrophages and which possessed long “dendrite-like” cytoplasmic processes capable of dynamic extension or retraction. From the time of this observation, it took nearly 5 years for Steinman's laboratory to develop a method for obtaining a pure population of dendritic cells. This accomplishment led to important discoveries that dendritic cells expressed high levels of major histocompatibility complex (MHC) proteins and that they were the most potent antigen presenting cells in the immune system for inciting a mixed leukocyte reaction (MLR) [3]. These observations led Steinman to predict that dendritic cells would “prove to be a critical accessory cell for the generation of many immune responses” [4]. Since Steinman's early discovery, dendritic cells identified in human blood were shown to uniquely capture and present antigens to T cells of the adaptive immune system [5, 6]. In the early 1990s, Steinman and his colleagues developed a method for production of human Langerhans dendritic cells from CD34^+^ progenitors with granulocyte macrophage colony stimulating factor (GM-CSF) and TNF-*α* [7]. Although this method increased the availability of DCs for future study, the amplification of DCs remained limited due to the scarcity of CD34^+^ progenitors in adult blood. This obstacle was overcome by the finding that large numbers of dendritic cells could be generated from peripheral blood mono nuclear cells (PBMCs) following treatment with GM-CSF and interleukin 4 (IL-4) [8]. This new monocyte-derived DC amplification system energized the study of dendritic cells by permitting the study of human DC functions in both healthy and diseased states. A detailed history of the discovery of dendritic cells is beyond the scope of this review and are covered in the cited reviews [3, 9–11].

Dendritic cells are the primary line of immune cell defense against pathogens and toxins that invade the body. Representing the innate portion of the immune system, dendritic cells recognize and destroy invading bacterial, viral, protozoan, and fungal pathogens and other foreign molecules that escape the body's passive defenses. In innate immunity, monocytes recruited into inflammatory sites differentiate into dendritic cells under the influence of Th1 cytokines [12]. The dendritic cell response comprises secretion of TNF-*α* and NO to aid in the clearing of pathogens [13]. The inflammatory cytokine TNF-*α* can bind to receptors on Gram-negative bacteria facilitating phagocytosis by macrophages [14]. Dendritic cells may also activate NK cells through both contact-dependent and contact-independent mechanisms [15]. When the pathogen load becomes excessive, dendritic cells act primarily as antigen presenting cells by migrating to the spleen or peripheral lymph nodes and delivering portions of the invading pathogens to lymphocytes of the adaptive immune system to amplify the immune response.

An increased awareness of the intricate relationship between dendritic cells and other cells of the adaptive immune system will be essential for understanding how immunological homeostasis is achieved and maintained. Further improvements in understanding how dendritic cells generate peripheral and central tolerance will be needed before more effective and safer therapeutic strategies can be constructed to prevent or reverse the process of autoimmunity. In central tolerance, dendritic cells residing in the thymus present antigens on MHCII and cross present self-antigens on MHCI receptors to bone marrow-derived T cells to stimulate apoptosis of potentially autoreactive T cells (negative selection) [16, 17]. Further, peripheral dendritic cells were shown to migrate to the thymus to induce clonal T cell deletion or development of T regulatory (Treg) cells [18, 19]. Although not yet demonstrated for all endogenous self-antigens, dendritic cells can induce peripheral tolerance through presentation of immunodominant antigens expressed at high levels [20]. Further, dendritic cells are thought to make a major contribution to peripheral tolerance by facilitating induction and/or maintenance of peripheral Treg cells [21]. Additional experimental evidence supports the role of dendritic cells in the induction of autoimmunity, reinforcing the notion that the activation state of dendritic cells is largely responsible for the induction of autoimmunity or tolerance [18]. Ablation of dendritic cells in mice with an autoimmunity-prone background was shown to ameliorate disease onset [22]. Together, this data shows that dendritic cells can stimulate the development of autoreactive T cells as well as generate immunological tolerance [23]. Later in this review, we discuss the origin of dendritic cell subsets and their respective functions in the initiation of inflammation as well as immune tolerance. Further, we review the involvement of dendritic cells in the induction of tissue-specific autoimmunity with comparisons to systemic autoimmunity to demonstrate how development of dendritic cell-based therapies for treatment of tissue-specific autoimmunity may become broadly applicable.

## 2. Dendritic Cell Origins and Functions

In order to efficiently encounter, process, and transport foreign antigens to lymph nodes for presentation to T cells, a variety of dendritic cell subsets exist that are specialized in morphology, function, and location [24]. Dendritic cells, like most cells of the immune system, comprise a number of groups or subsets that are distinct either in origin, function, or both. Although classification of DC subsets may vary, this review focuses on the four major categories of dendritic cells currently recognized: (1) conventional dendritic cells (cDCs), (2) Langerhans cells, (3) plasmacytoid dendritic cells (pDCs), and (4) monocyte-derived dendritic cells (moDCs). Due to differences in phenotype, an alternative subset of dendritic cells, the myeloid DCs (mDCs), are also included. The myeloid DC subset consists of both cDCs and moDCs.

### 2.1. Dendritic Cells Originate in the Bone Marrow

Dendritic cell hematopoiesis was originally established in mice and the corresponding DC subsets were identified in humans. The origin of dendritic cells begins in the bone marrow with CD34^+^ FLT3^+^ common myeloid progenitor (CMP) cells [25]. The CMPs differentiate into restricted macrophage dendritic cell progenitors (MDP). The MDPs branch to create two separate dendritic cells lineages: the monocyte and the common dendritic cell progenitor (CDP) [24]. Monocytes circulate in the periphery and during inflammation, differentiate into CD11c^+^, CD11b^+^, and MHCII^+^ moDCs [26]. The CDPs differentiate in the bone marrow into plasmacytoid DCs and into pre-DCs which give rise to conventional DCs in peripheral tissues [24]. The only dendritic cells that do not originate in the bone marrow are the Langerhans cells. The Langerhans subset is derived from a local Ly6C^+^ myelomonocytic precursor in the skin that originates from macrophages during early embryonic development [27]. Petvises and O'Neill provide a complete review of dendritic cell hematopoiesis [28].

### 2.2. Conventional DCs and Langerhans Cells

Conventional dendritic cells are a highly specialized DC subset that is efficient in antigen processing and presentation. This major group of dendritic cells can be categorized as either migratory or lymphoid tissue resident DCs. Migratory dendritic cells develop from precursors in both lymphoid and nonlymphoid tissues but are not found in the spleen [29]. After taking up and processing antigens from resident tissues, cDCs migrate to the peripheral draining lymph nodes via afferent lymph vessels to present sequestered antigens to naïve T cells. The migration of cDCs is markedly amplified during inflammatory conditions. In contrast, lymphoid tissue resident dendritic cells are located in the spleen, thymus, and lymph nodes. In comparison with the migratory DC subset, lymphoid tissue resident dendritic cells do not migrate but rather develop from dendritic cell precursors already residing in lymphoid tissues [30]. During immunological homeostasis, cDCs remain immature, have high endocytic capacity, and synthesize low levels of MHC relative to other dendritic cell subsets [31]. Due to their predominant location in the spleen, lymphoid tissue resident dendritic cells are well suited to sample antigens transported by the blood. Langerhans cells function in a manner similar to migratory conventional DCs. They reside in the skin and can be identified by the expression of the monocyte/macrophage and endothelial cell differentiation antigen Ly6C [32]. Their function is to capture, identify, and present antigens from the external environment to naïve T cells. Both cDCs and Langerhans cells are necessary for the maintenance of immunological homeostasis, with their major functions occurring during the steady state.

### 2.3. Plasmacytoid Dendritic Cells

Plasmacytoid dendritic cells arise from lymphoid progenitors and are broadly distributed throughout the body. In humans, this DC subset can be identified through expression of immunoglobulin-like transcript 7 (ILT7) and CD45R [33]. Plasmacytoid dendritic cells are generally quiescent, but when stimulated, they secrete large amounts of type 1 interferons which induce antiviral responses in other immune cells [34]. Plasmacytoid dendritic cells preferentially express intracellular TLRs including TLR7 and TLR9. These toll-like receptors bind pathogen nucleic acids, especially motifs rich in CpG, and play a major role in pDCs response to viral infections [35]. Because pDCs have low antigen presentation capabilities, their role in promoting adaptive immunity remains relatively unclear [36]. However, pDCs are known to play a major role during inflammation as opposed to cDCs which are more involved in the maintenance of immunological homeostasis.

### 2.4. Monocyte-Derived Dendritic Cells (Myeloid DCs)

Monocyte-derived dendritic cells arise from myeloid progenitors and are crucial for immune responses because they provide a pool of antigen presenting cells that can effectively initiate an adaptive immune response following the onset of infection. During inflammation, it is well known that circulating monocytes express receptors for GM-CSF, M-CSF, IL-4, and other differentiation and chemoattractant molecules that recruit them to the site of inflammation and differentiate them into moDCs. Monocyte-derived dendritic cells are highly potent in antigen processing, presentation, and cross presentation [26]. They are CD11c positive and express high levels of MHC II in comparison with other DC subsets. The function of moDCs is similar to cDCs because they both process foreign antigens from tissues and migrate to the nearest draining lymph nodes where they present the antigens to naïve T cells. Upon activation, the lifespan of all DC subsets is relatively similar, as opposed to the length of time DCs reside in tissues during their immature state [37]. The major responsibility of moDCs and pDCs is to incite an adaptive immune response through activation of T cells that can resolve inflammation and return the immune system to immunological homeostasis. The interaction of moDCs and pDCs with autoreactive T cells can either induce autoimmune dysfunction or initiate tolerance.

## 3. The Role of Dendritic Cells in Autoimmunity

Dendritic cells have well-defined roles in both innate and adaptive immunity. However, it is their ability to link the innate and adaptive immune system that confers them a role in autoimmunity. In adaptive immunity, DC-secreted factors affect IgA production by B cells [38]. Most importantly, DCs transfer innate signals to the adaptive immune system by priming naïve T cells, by stimulating Th1, Th2, and Th17 responses, by cross presentation of antigens to CD8^+^ T cells, and by regulating T cell differentiation [39–42]. The breakdown of dendritic cell functions is considered to be the driving force behind the onset of tissue specific autoimmunity.

### 3.1. Dendritic Cell Identification of Pathogens

Dendritic cell subsets differ in their specialized functions, including the location of activity, cytokine profiles, types of antigens detected, migratory, or tissue resident status and presence during immunological homeostasis or during inflammation. However, the common function of these dendritic cell subsets is the communication of inflammatory or immunosuppressive signals among the innate and adaptive cells of the immune system. This function requires two main steps. The first is the identification of antigens. The second step is the presentation of antigens along with the appropriate secondary signals required to induce an adaptive T cell response. Dendritic cells identify antigens via pathogen-associated molecular patterns (PAMPS) or damage-associated molecular patterns (DAMPs). Comprised of molecules common to a variety of organisms but absent in the host, PAMPS are an exogenous signal to dendritic cells for infection. For example, viral envelope proteins and ssDNA from viruses, lipopolysaccharide and flagellin from bacteria, zymosan from fungus, and profilin from* Toxoplasma gondii* are PAMPs which are recognized by specific receptors on dendritic cells. In comparison, DAMPs are endogenous “danger” signals from within cells. Release of ATP, DNA, or uric acid can be a warning sign to dendritic cells for stress, microbial invasion, or necrotic cell death such as in the case of cancer [43]. Dendritic cells detect PAMPS and DAMPs through several classes of surface and intracellular receptors called pattern recognition receptors (PRRs). These classes include the receptor for advanced glycation end products (RAGE), RIG-I-like receptors (RLRs), NOD-like receptors (NLRs), and Toll-like receptors (TLRs).

Certain DAMPS such as heat shock and S100 proteins are recognized by RAGE. Other DAMPs, such as ATP and uric acid activate NLRs and induce the formation of inflammasomes which trigger the downstream secretion of inflammatory cytokines IL-1*β* and IL-18. All immunogenic nucleotides bind RLRs which need subsequent recognition to induce a signaling response within the dendritic cells. Lastly, TLRs, the most common of the PRRs, recognize a variety of PAMPs and DAMPs and are largely implicated in autoimmune disease. Surface TLR4, TLR5, and heterodimers of TLR1/2 and TLR2/6 all recognize bacterial membrane components while TLRs 3, 7, 8, and 9 are located within endosomes and recognize immunogenic nucleotides [43]. The high expression of TLR- 2 and TLR-4 on mDCs and TLRs 7 and 9 on pDCs may have a role to play in tissue-specific autoimmunity where molecular mimicry or autoantibodies to nucleic acids is a potential underlying mechanism.

### 3.2. Dendritic Cell Presentation of Antigens to T Cells

Once a dendritic cell has encountered an antigen, it processes and transfers this information to T cells. Mature DCs interact with T cells through (1) dendritic cell MHC-II/antigen complex interactions with the T cell receptor, (2) dendritic cell CD86/CD80 costimulation of T cell CD28, and (3) dendritic cell cytokine signaling to T cells. Because the extent of DC activation determines whether interaction with a T cell will induce tolerance or immunity, antigen presentation in the presence of inflammatory signals results in immunity while Ag presentation during the steady state after results in tolerance [44]. During inflammation, dendritic cells can induce naïve T cell (Th0) differentiation or activate memory T cells. Upon dendritic cell maturation, naïve CD4 T cells can differentiate into memory and Th1, Th2, and Th17 effector cells associated with autoimmunity. Naïve CD8 T cells differentiate into cytotoxic T lymphocytes and CD8 memory cells. During immunological homeostasis, DC interaction with T cells generates and maintains a population of Tregs. Under conditions of autoimmunity, autoantigens which should induce tolerance during homeostasis generate an inflammatory response. In general, the role of DCs during the development of autoimmunity is to induce autoreactive CD4^+^ and CD8^+^ proinflammatory T cell differentiation rather than immunosuppressive Treg development or autoreactive T cell anergy.

### 3.3. Role of Dendritic Cells in the Onset and Prevention of Autoimmune Responses

Dendritic cells play a critical role in both the prevention and onset of autoimmune responses. In the nondiseased state, DCs are responsible for the induction and maintenance of tolerance towards self-antigens. The induction of T cell tolerance is dependent on whether a DC is tolerogenic or immunogenic during autoantigen presentation, and also on the contribution of autoreactive T cells that may escape from the thymus. Although the continuously developing T cell repertoire is rigorously screened in the thymus to develop central tolerance, autoreactive T cells which may escape apoptosis in the thymus are poised to induce autoimmunity unless regulated by dendritic cells in the periphery [45]. Normally, presentation of a self-antigen to an autoreactive T cell results in T cell anergy, deletion, activation, or induction of Treg cells [46].

There are several factors that influence whether a DC will induce tolerance when presenting self-antigens including but not limited to activation state and method of antigen capture/antigen source. The activation state of a DC is crucial to the fate of the T cells with which it interacts. Typically, tolerogenic DCs express low levels of costimulatory molecules (CD80, CD86), generate increased secretion of IL-10 and TGF-*β*, and reduce secretion of proinflammatory IL-12, IL-1, IL-6, and TNF cytokines. In addition to these hallmarks of tolerogenic DCs, the secretion of IL-2 and a variety of enzymes such as retinaldehyde dehydrogenase-2 and indolamine 2,3 dioxygenase are involved in the suppression of Th1-mediated autoimmunity in addition to the induction of Treg cells [46–48].

The DCs ability to capture process and present antigen is also integral to defining an immunogenic or tolerogenic phenotype. Apoptotic cells in comparison with necrotic cells are tolerogenic. Tam receptor kinases (Tyro3, Axl, Mer) present on apoptotic cell membranes inhibit TLR and cytokine-induced signaling cascades, therefore preventing immunogenicity of the autoantigens presented [49]. In addition, TGF-*β* is associated with Treg induction and is secreted from DCs upon uptake of apoptotic cells [50, 51]. Posttranslational modification of proteins also helps determine the immunogenicity of an autoantigen. Dendritic cells can capture highly glycosylated proteins through C-type lectin receptors (CLRs) present in the plasma membrane. Because CLRs are involved in the clearance of multiple soluble self-antigens, this posttranslational modification is tolerogenic [52]. Alternatively, acetylation of proteins can produce neoantigens to which the immune system has not been tolerized [53].

All of these factors are in place to ensure a tolerogenic phenotype in DCs during presentation of self-antigens. However, these mechanisms to prevent the human immune system from recognizing self-antigens may occasionally malfunction. In general, this malfunction can involve self-antigen presentation in the presence of danger signals, therefore breaking tolerance. For each autoimmune disease, different mechanisms for DC initiation of autoimmunity are suggested; nonetheless, they still remain unclear. For example, in the case of type 1 diabetes, genetic abnormalities in DC subsets and viral infection have been linked as initiators of autoimmunity [54]. NOD mice have increased CD11b^+^ cDCs which have been demonstrated to be responsible for the presentation of type 1 diabetes autoantigens to autoreactive T cells [55, 56]. In the diseased state, these cDCs cross-present islet antigens to autoreactive CD8 T cells and secrete increased IL-12, TNF*α*, and IL-1 [54]. The breach of tolerance induced by cDCs is speculated to be regulated by pDCs [54]. In some cases, certain viral tropisms induce the secretion of type 1 interferons by binding TLRs within pDCs. A localized type 1 interferon response within the pancreas can activate autoreactive T cells and act as a danger/maturation signal to resident cDCs, therefore promoting presentation of steady state antigen but in a proinflammatory context [57]. This immunogenic presentation of islet autoantigens to CD8 T cells may allow for the homing of cytotoxic T cells to the islets, destruction of the target cells, and the perpetuation of autoimmunity.

Systemic lupus erythematosus is an example of a systemic autoimmune disease for which there are models describing the chain of events linking DC activation to an adaptive immune response. In genetically susceptible individuals, immune complexes consisting of nucleic acid-associated autoantigens and autoantibodies which are internalized by pDCs via Fc*γ*RIIa receptors activate internal TLRs [58]. This TLR activation, just like a response to viral infection, induces the secretion of type 1 interferon. Type 1 interferons induce maturation of cDCs, increasing the expression of MHC class I and II, costimulatory molecules, and chemokines and chemokine receptors [59]. This scenario allows for the uninhibited presentation of autoantigens by proinflammatory DCs and thus the expansion of autoreactive T cells.

For other autoimmune diseases such as multiple sclerosis, the chain of events linking DC activation by PAMPs or DAMPs to induction of autoreactive T cells is relatively unknown and is an important area for future research. In summary, the induction of autoimmunity by DCs requires excessive production of PAMPs or DAMPs to switch DC presentation of autoantigens from tolerogenic to immunogenic, usually on a genetically susceptible background [60]. The switch of the DC to a proinflammatory state disrupts tolerance by activating and inducing differentiation of autoreactive T cells via TCR ligation and cytokines such as IL-2, IL-12, IL-6, type 1 interferons, and TGF-*β*. In the following segments, we will review current evidence for dendritic cell-induced autoimmunity in tissue-specific autoimmune diseases. We will also identify potential dendritic cell targeted therapies and discuss their mechanisms of action.

## 4. Dendritic Cell Stimulation of Tissue-Specific Autoimmunity

Tissue-specific autoimmunity requires the release of specific autoantigens characteristic of a given tissue or organ that can be recognized by dendritic cell PRRs. Prototypical tissue-specific autoimmune disease examples presented in this review are type 1 diabetes (T1D) and multiple sclerosis (MS), and for comparison examples of systemic autoimmune diseases discussed are rheumatoid arthritis (RA) and systemic lupus erythematosus (SLE). The pancreatic *β*-cell is one of the most specialized cells in the body and is central to type 1 diabetes onset. In addition to the production, storage, and secretion of the peptide hormone insulin, the *β* cell is capable of sensing and responding to changes in blood glucose levels [61]. During the earliest stages of T1D research, specific islet autoantigens signaling diabetes onset were discovered through their recognition by islet cell autoantibodies (ICAs). Since identification of ICAs in 1976, their *β*-cell protein targets were only slowly revealed [61, 62]. With the exception of insulin as an obvious T1D candidate autoantigen, it was not until 1990 that the 64 kDa glutamic acid decarboxylase (GAD) protein was discovered to be an important secondary antigen for T1D development [61, 63].

Multiple sclerosis (MS) is a devastating inflammatory disease of the brain and spinal cord resulting from autoimmune attack against antigens in the central nervous system [64]. Proinflammatory T cell responses to the major myelin proteins, myelin basic protein (MBP), and proteolipid protein (PLP) are considered to be important for the development of MS [65]. However, DC-mediated autoimmune responses to other minor myelin antigens such as myelin-associated glycoprotein (MAG) and myelin oligodendrocyte glycoprotein (MOG) may also play a significant role in disease initiation or progression [65].

**Figure 1:**
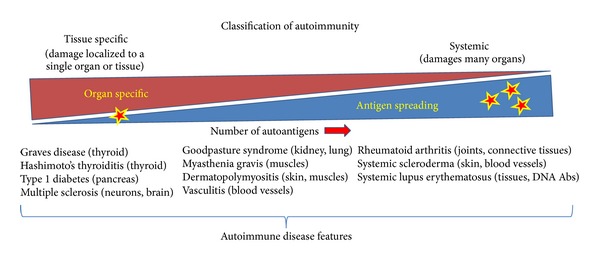
Characteristics of tissue-specific and systemic autoimmune disorders. Tissue-specific autoimmunity originates in a specific tissue within an individual organ usually initiated by a single autoantigen. Dendritic cell MHC presentation of this antigen to cognate autoreactive T cells amplifies an adaptive immune response that kills the antigen producing cells. The death of these cells releases a variety of cellular antigens that amplify the inflammatory immune response (antigen spreading), represented here as stars. In contrast, systemic autoimmunity autoantigens may originate independently within different tissues or organs in the body, for example, connective tissues in rheumatoid arthritis (RA) and systemic lupus erythematosus (SLE). Thus, antigen spreading can originate from multiple tissues in a variety of affected organs at different times (multiple stars), leading to diverse inflammatory disease progression from patient to patient. Several tissue-specific autoimmune diseases and their organs of origin are listed (left). Autoimmune diseases originating in several organs (center) and systemic autoimmune diseases originating independently in many organs throughout the body are indicated (right).

Unlike organ-specific autoimmune diseases, a major challenge exists in finding individual representative antigens for initiating the onset of systemic autoimmunity (Figure 1). This difficulty is based on observations by many investigators that the onset of systemic autoimmunity may be initiated at different times and locations in the connective tissues of rheumatoid arthritis (RA) and systemic lupus erythematosus (SLE) patients. Further, the problem of progressive antigen spreading can further complicate this issue. In confirmation that tissue specificity underlies systemic autoimmunity, it was recently shown that hyperactivation of MyD88-adapter-dependent signaling in DCs is sufficient to drive pathogenesis of lupus-like autoimmunity [66]. This result emphasizes that dysregulation of dendritic cells alone can lead to autoimmunity.

Systemic lupus erythematosus (SLE) is an autoimmune disease that progressively invades many tissues throughout the body and is frequently characterized by the formation of anti-nuclear and anti-chromatin antibodies. These autoantibodies are generated in response to aberrant apoptosis and decreased clearance of apoptotic cells which increases the abundance of apoptotic cell blebs containing chromatin. In addition, the chromatin can be modified during apoptosis further increasing its immunogenicity [67]. Interestingly, mDCs can take up, process, and present chromatin found in these apoptotic blebs to T cells. Dendritic cell presentation of this modified chromatin stimulates activation of autoreactive T helper cells, leading subsequently to the formation of autoantibodies by autoreactive B cells. The deposition of immune complexes formed by anti-chromatin autoantibodies and modified chromatin on cell basal membranes stimulates a local inflammatory response [68]. Further, autoreactive T cells that bind nuclear antigens such as DNA and histones or small ribonucleoproteins including Smith (Sm) antigens and U1 and heterogeneous ribonucleoprotein (hnRNP) A2 to their T cell receptors were shown to be associated with SLE development [69]. Taken together, this data suggests that chromatin, Smith antigens, and ribonucleoproteins are potential autoantigen candidates for development of SLE.

Rheumatoid arthritis (RA) is another well-known systemic autoimmune disease that affects connective tissues throughout the body. RA-related autoantibodies demonstrate reactivity to citrullinated proteins and peptides designated as anti-citrullinated protein antibodies (ACPA) [70]. The process of citrullination removes positive charges from the antigen via replacement of arginine with citrulline. The alteration in charge can modify secondary and tertiary protein structure, thereby increasing the binding affinity to MHC receptors [71]. The citrullinated proteins, now altered in structure from normal body proteins, may be recognized by the immune system as foreign and thus, potential autoantigens integral to the development of RA. In agreement with this concept, autoreactivity towards a variety of self-proteins has been associated with the onset and progression of RA. Several of the antigens described are joint-derived proteins, such as type II collagen and human cartilage-derived glycoprotein HCgp39 [72]. These experimental findings suggest the possibility of treating both tissue-specific and systemic autoimmune diseases by targeting several specific autoantigens characteristic of disease development.

### 4.1. Dendritic Cell Stimulation of Type 1 Diabetes Autoimmunity

Type 1 diabetes is a juvenile onset form of diabetes resulting from autoimmune destruction of insulin-producing *β* cells in the pancreatic islets of Langerhans. Type 1 diabetes can be treated in a palliative fashion with exogenous insulin injection. However, the increasing prevalence of this disease, its progressive complications, and the lack of effective curative and preventive strategies demand a significant research effort to identify promising therapies capable of restoration of immunological tolerance. At present, no effective, safe, and economical treatment exists to control the onset and progression this life-long debilitating disease [73]. For this reason, the nonobese diabetic (NOD) mouse, a widely used animal model presenting the dominant symptoms of human T1D autoimmunity, is frequently used for studying the mechanisms underlying T1D onset and progression [74].

Based on the presence of specific autoantigens known to elicit diabetes onset, T1D has been identified as a model prototypic tissue-specific autoimmune disease. Hyperglycemia develops in T1D when insulin presenting DCs encounter naive insulin reactive T cells in the periphery. During this interaction, the DCs guide autoreactive T cell differentiation into inflammatory effector cells that arrest insulin production by inducing *β*-cell apoptosis. Little is known concerning the kinetics and phenotype of DCs in the NOD mouse pancreas during T1D development. While peri-islet accumulation of cDCs can be observed in NOD mice as early as 4 weeks of age, pDCs were shown to accumulate around the islets of Langerhans beginning later at 10 weeks of age. Peri-islet dendritic cell accumulation was found to be concomitant with the influx of lymphocytes [75]. Ablation of total DCs in NOD mice led to loss of CD4^+^ T cell activation, insulitis, antibody production, and pancreatic infiltration with proinflammatory Th1/Th17 cells [76]. The authors further demonstrated that reintroduction of mDCs to the DC ablated mice induced insulitis and diabetes. Together, these results demonstrate that DCs are key players in the onset of tissue specific autoimmunity.

The state of dendritic cell activation is critical for determination of their function as tolerogenic or inflammatory DCs. An important feature of tolerogenic DCs is their ability to secrete the immunoregulatory cytokine TGF-*β* as well as the anti-inflammatory cytokine IL-10 which can suppress T cell responses by inhibiting T cell secretion of IL-2 and IFN-*γ* [77]. Presence of the anti-inflammatory cytokine IL-10 was shown to control a number of different immune cell types implicated in the inflammatory response, including DCs [78]. In addition, IL-10 was shown to upregulate the expression of tolerogenic molecules like ILT3 and ILT4, allowing them to minimize immune responses and induce Treg morphogenesis [79, 80]. During the immunological steady-state also referred to as homeostasis, DCs were shown to secrete high levels of IL-10 that can modulate activation of neighboring myeloid DC and promote* de novo* induction of tolerogenic DCs. In view of their pivotal role in regulating T cell immunity, dendritic cells could be expected to alter the balance between pathogenic T cells and Tregs in type 1 diabetes. Studies in the NOD mouse have shown that mDCs can exhibit a hyper-inflammatory phenotype [81, 82]. Specifically, NOD mouse mDCs have been shown to generate an elevated capacity for stimulation of T cells and secretion of proinflammatory cytokines such as IL-12. Further,* in vitro* studies have shown that maturation of moDCs is inhibited in the presence of exogenous IL-10. The resultant DCs become capable of inducing T cell anergy and Treg cell differentiation [83].

### 4.2. Dendritic Cell Stimulation of Encephalomyelitis (Multiple Sclerosis)

Multiple sclerosis is a chronic inflammatory disease of the central nervous system (CNS) associated with an autoimmune response against components of myelin, including myelin basic protein [84]. Experimental autoimmune encephalomyelitis (EAE), the animal equivalent of MS, is the prominent tool by which researchers studying MS have investigated the disease process. Various versions of the model are under investigation to study the mechanisms of immunopathogenesis and new treatments for MS. New treatment strategies are frequently tested in a chronic EAE mouse model, and when successful, the treatment can be considered for human therapy.

Over the last two decades, the knowledge of immunopathogenesis in MS has grown to define MS as a multifocal demyelinating disease mediated by an autoimmune response to several self-antigens. Although neurological deficits in MS may be the result of a combined cellular and humoral autoimmune attack on the myelin sheath, MS has long been considered a predominantly T cell-mediated autoimmune disease [85]. However, it is clear that T cells are not the only immune cell type involved in MS and EAE disease pathogenesis. The characterization of DCs during the course of EAE development indicates that different DC subsets serve distinct functions. For example, conventional DCs are involved in disease development, while plasmacytoidDCs that produce interferons (IFN) are important in the development of Treg cells and disease resolution [86]. In MS, recent experimental findings highlight the upregulation of TLR7 mediated by interferon-*β* (IFN-*β*) in pDCs. Upregulation of TLR7 in pDCs and consequently increased activation of pDCs by TLR7 ligands could be considered novel immunoregulatory mechanisms for IFN-*β* [87]. A critical role for IFN-*β* was demonstrated in a recent finding in which the proinflammatory cytokine induces expression of the IL7R*α* receptor [88]. Expression of IL7R*α* is unequivocally associated with susceptibility to MS and was shown to be increased in mDCs in a haplotype-dependent manner in response to increases in IFN*β*.

Active participation of DCs in the pathology of MS is supported by their presence and activation in the CNS of MS patients [89]. Myeloid and plasmacytoid dendritic cells are present in cerebrospinal fluid in noninflammatory neurological diseases and elevated in multiple sclerosis and in acute monosymptomatic optic neuritis [90]. Particular emphasis has been given to the study of pDCs involvement based on their importance in stimulating or inhibiting effector T cells in MS [86].

In addition to the presence of DCs in cerebrospinal fluid (CSF) and CNS lesions in MS patients, both phenotypic and functional impairments have also been observed to be dependent on DC subsets and MS subtypes. Circulating cDCs expressing upregulated levels of costimulatory molecules and proinflammatory cytokines stimulate proinflammatory cytokine secretion by effector T cells. Their infiltration into the inflamed brain can be attributed to upregulation of C-C chemokine receptor 5 (CCR5) [91]. Ambivalent functions of pDCs have been observed in EAE. It was suggested that pDCs promote priming of autoimmune Th17 lymphocytes in EAE, whereas depletion of pDCs prior to induction of the disease decreases its severity [92]. Abundant expression of TLR9 in pDCs appears to be important in the pathogenesis of EAE. Activation of APCs through TLR9 can overcome tolerance and precipitate EAE while TLR9 knockout mice show a decreased susceptibility to EAE [93, 94].

### 4.3. The Role of Dendritic Cells in Autoimmunity-Mediated Epitope Spreading

Antigen or epitope spreading is a process in which immunoreactive segments of a protein (epitopes) distinct from and non-cross-reactive with initial disease-inducing antigen epitopes become targets of a progressive inflammatory immune response. The phenomenon of epitope spreading has been defined in experimental and naturally occurring inflammatory responses as a consequence of acute or persistent infection characteristic of the chronic tissue destruction that occurs during autoimmune disease progression [95]. Epitope spreading has been described in different models of autoimmunity, including T1D and EAE. Epitope spreading may occur as increasing numbers of cells of the affected tissue or organ undergo necrosis or necroptosis following the initial acute immune response. The progress of inflammatory cell death may allow for the capture, processing, and presentation by DCs of an increasing number of cellular antigens. Thus, the number of autoantigens presented in this progressive inflammatory state would increase and tolerance to these autoantigens would be lost.

In type 1 diabetes, pancreatic resident DCs are generally the first cells of the immune system to process *β*-cell autoantigens, and by promoting autoreactivity, they play a major role in the onset of pancreatic inflammation (insulitis) [96]. Protection from the onset of autoimmune disease may be induced by the introduction of candidate autoantigens (in the case of T1D: proinsulin, insulin, heat shock protein 60, or glutamic acid decarboxylase) considered to be the initial major offending autoantigens [96]. Results of mechanistic studies have confirmed that downregulation of the immune response specific to the disease autoantigen can rapidly extend to other candidate autoantigens [97, 98]. These experimental findings suggest that therapeutics may not need to be targeted toward an ever increasing number of autoantigens found both in tissue-specific and systemic autoimmunity but may be effective through targeting one of the major autoantigens found during the acute phase of disease onset. Because the specific role of DCs in epitope spreading in type 1 diabetes has yet to be elucidated, epitope spreading remains an obstacle in autoantigen targeted therapy.

Dendritic cells have been shown to be particularly critical in epitope spreading in the experimental autoimmune encephalomyelitis (EAE) model. In EAE, reactivity to myelin epitopes generated during the initial clinical episode of relapsing EAE (R-EAE), for example,* epitope spreading*, plays a major role in the mediation of further clinical relapses [99–102]. Determination of the capacity for antigen delivery by antigen-presenting cell (APC) populations purified from the central nervous system (CNS) of mice with established R-EAE shows that peripherally derived CD11b^+^CD11c^+^CD45^hi^ myeloid dendritic cells (mDCs) are efficient in presentation of endogenous myelin antigens that stimulate the differentiation of both preactivated effector myelin-specific T cells and naïve T cells [103]. The mDCs, which drive epitope spreading, preferentially polarize pathogenic Th17 responses that correlate with their enhanced expression of cytokines TGF-beta1, IL-6, and IL-23 [102]. In the same R-EAE model, it was shown that DCs, macrophages (F4/80^+^CD45^hi^), and microglia (F4/80^+^CD45^lo^) activate a PLP139-151-specific T helper cell line [103]. The data from this study and others suggest that DCs presenting CNS antigens migrate from CNS tissue to prime encephalitogenic myelin-reactive T cells in lymphoid organs, thereby inducing antigen spreading and recruitment of T cells into the inflamed CNS [104].

In rheumatoid arthritis (RA), autoantibodies targeting several innate immune cell ligands including citrullinated histones, fibrinogen, and biglycan have provided insights into the earliest autoantigen targets and potential mechanisms responsible for the onset and development of RA autoimmunity. In addition, expansion of anti-citrullinated protein antibodies (ACPA) has strongly predicted increases in many inflammatory cytokines in RA including TNF-*α*, IL-6, IL-12p70, and IFN-*γ*. Thus, it was observed that the preclinical phase of RA can be characterized by accumulation of multiple autoantibody specificities that reflect the process of antigen spreading [105].

Autoantibodies targeted against nuclear components are a characteristic feature of SLE. Due to disturbed apoptosis and/or an insufficient clearance of apoptotic debris, the nucleosome is a major source of autoantigens in SLE patients [106]. Recent studies have identified apoptosis-induced acetylation of histone H2BK12 as a target for autoantibodies in SLE. Since anti-H2BK12ac reactivity was found mainly in prediseased lupus mice, this epitope seems to be important in the early phase of antichromatin autoimmune responses leading to subsequent epitope spreading to unmodified histone H2B [107]. Plasmacytoid DCs can internalize immune complexes formed by anti-chromatin antibodies and present autoantigen to B cells [59]. This presentation, under proinflammatory conditions, could induce B cell differentiation to plasma cells or induce the secretion of more autoreactive Abs which could form more immune complexes and ultimately induce a feed-forward proinflammatory loop that might contribute to antigen spreading in SLE. Understanding antigen spreading in autoimmunity will be crucial for the design of effective DC-interfering therapeutics; therefore, additional research will be needed to further define the role of DCs in autoimmunity-mediated antigen spreading.

## 5. Suppression of Tissue-Specific Autoimmunity through DC-Interfering Therapeutic Strategies

**Figure 2:**
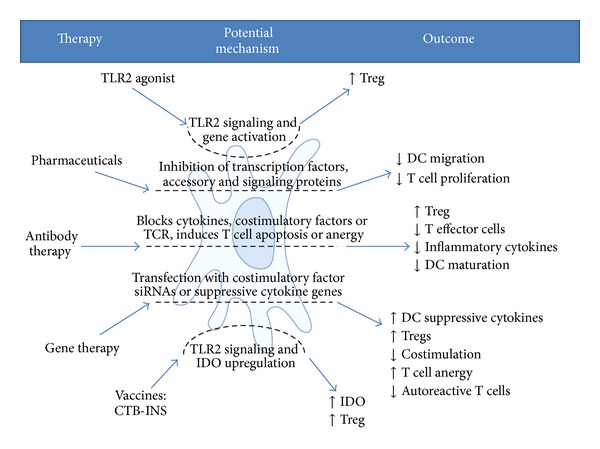
Potential mechanisms responsible for dendritic cell-mediated suppression of autoimmunity. (Left) Therapeutic strategies for DC-mediated therapy for tissue-specific autoimmunity. (Center) Molecular interactions by which these therapeutic strategies may function to suppress autoimmunity. (Right) Immune cell outcomes following the therapy that may stimulate immunological tolerance.

A variety of molecular and cellular strategies for suppression of tissue-specific autoimmunity are currently under development (Figure 2). These strategies include agonist or antagonist mediated interactions, pharmaceuticals, cytokine targeted antibody therapies, and immunosuppressive vaccines. Mechanisms by which DCs may be able to mediate the suppression of tissue-specific autoimmunity are discussed below.

### 5.1. TLR2 Agonists

How dendritic cells become activated and the nature of the activation state remains a question for further investigation. However, it has been shown that Toll-like receptor 2 (TLR2) can recognize molecular motifs in atypical LPS, peptidoglycan, lipoteichoic acid, lipoproteins, and lipopeptides [108]. More recently, it was shown that chronic administration of the TLR2 agonist Pam3CSK4 could prevent diabetes onset in NOD mice by inducing DC-mediated tolerance [109]. Further corroborating these experimental findings, additional research has shown that TLR2 signaling can modulate immune regulation and alter the progression of autoimmunity in the NOD mouse. These experimental results suggest a role for TLR2 in enhancement of CD4^+^CD25^+^ Treg proliferation both in a naïve T cell context and during viral infection to provide increased protection against development of autoimmune diabetes [108]. Treatment of prediabetic mice with a synthetic TLR2 agonist diminished the onset of T1D and increased the number and function of CD4^+^CD25^+^ Tregs, thereby conferring tolerogenic properties to DCs. The ligation of dendritic cell TLR2 was also shown to increase their capacity for autoimmune disease prevention and to promote the proliferation of Tregs [108].

### 5.2. Pharmaceuticals

#### 5.2.1. Inhibitors of Calcineurin, Cyclosporine, and Tacrolimus

Cyclosporine and tacrolimus, complex nonantibiotic macrolide compounds isolated from soil fungi and bacteria, are widely used as immunosuppressive agents following solid organ transplantation. These immunosuppressant drugs were shown to dampen the inflammatory activity of the immune system by interfering with the activity and growth of T cells [110]. Cyclosporine and tacrolimus cause immune suppression by binding cytoplasmic cyclophilin and FK-binding proteins, respectively. Generation of these protein complexes induces binding to calcineurin, therefore blocking calcineurin's activation of the T cell transcription factor NFATc. This blockade results in inhibition of secretion of the inflammatory cytokine IL-2. Consequently, T-cell proliferation is suppressed as evidenced by the ability of cyclosporine treated myeloid DCs to suppress the proliferation of allogenic peripheral blood mononuclear cells (PBMCs) [111]. Similarly, stimulation of memory CD8^+^ T cells by DCs was impaired by cyclosporine pretreatment. From this study, it was concluded that cyclosporine differentially alters the function and phenotype of mDCs leading to a partially impaired capacity to stimulate the activation of allogenic and autologous T cells. In another study, it was found that cyclosporine A (CsA) impaired the migration of mouse bone marrow-derived DCs toward macrophage inflammatory protein-3beta (MIP-3beta) and induced them to retain responsiveness to MIP-1*α* after lipopolysaccharide (LPS) stimulated DC maturation* in vitro*. Administration of CsA* in vivo* was shown to inhibit the migration of DCs out of skin and into the secondary lymphoid organs [112]. Further, it was also shown that cyclosporine suppresses *β*-cell autoimmunity and rescues islet *β*-cell function [112]. However, this study indicates that the therapeutic effect of CsA is sustained only with continuous cyclosporine administration, which unfortunately is associated with significant adverse effects. Cyclosporine was shown to downregulate DC synthesized inflammatory cytokines IL-2 and IL-12, suggesting this class of inhibitory molecules may have an important role in the regulation of DC-mediated inflammatory immune responses [113].

#### 5.2.2. Sirolimus (Rapamycin)

Sirolimus is a complex organic molecule isolated from bacteria that exerts a suppressive effect on the immune system but acts differently from the calcineurin inhibitors cyclosporine and tacrolimus [114]. Sirolimus was shown to inhibit responses to the inflammatory cytokine IL-2, thereby blocking T and B cell activation. In contrast to cyclosporine and tacrolimus, sirolimus inhibits IL-2 secretion by binding to the cytosolic protein FK-binding protein 12 (FKBP12) [115]. The sirolimus-FKBP12 complex blocks the mammalian mTOR pathway through direct binding to the mTOR Complex1 (mTORC1) [116]. Resistance to maturation and tolerogenic properties of DCs were shown to be supported and preserved by conditioning with sirolimus [117]. The ability of sirolimus to suppress DC activation suggests that sirolimus/rapamycin-based therapeutic strategies may be effective for the inhibition of tissue-specific autoimmunity.

#### 5.2.3. Anti-Delta-Like Ligand 4 (Dll4)

The Notch signaling pathway is a highly conserved cell signaling system present in most multicellular organisms [118]. The Notch family of proteins is transmembrane proteins with extracellular epidermal-like growth factor (EGF) domains. Ligand proteins binding to the EGF domains induce proteolytic cleavage and release of the intracellular domain, which enters the cell nucleus to modify gene expression [119]. Interaction between Notch receptors and their ligands represents an evolutionarily conserved signaling pathway important for cell fate commitment in hematopoiesis and thymus development [120–122]. A new addition to the Delta family of Notch ligands, named Delta-like ligand 4 (Dll4), is predicted to encode a membrane-bound ligand. DII4 is characterized by an extracellular region containing several EGF-like domains and a Delta/Serrate/LAG-2 (DSL) domain required for receptor binding [123]. Studies have shown that Dll4 is an essential and nonredundant Notch1 receptor ligand and its specific inactivation on thymic epithelial cells (TECs) leads to a block in T cell development accompanied by ectopic appearance of an alternative B cell lineage within the thymus [120, 124].

In a recent finding, an anti-Delta-like ligand 4 (Dll4)-Notch signaling treatment was shown to fully prevent T1D in NOD mice via a Treg cell-mediated mechanism. Further, this treatment inhibits CD8^+^ T cell pancreatic islet infiltration. Treatment with anti-Dll4 was shown to convert CD4^−^CD8^−^c-kit^+^CD44^+^CD25^−^ (DN1) T cell progenitors to immature DCs that induce* ex vivo* differentiation of naive CD4^+^ T cells into Treg cells. A single injection of anti-Dll4 antibody was shown to reverse established T1D [120]. These results identify Dll4-Notch as a novel pathway that may be important for regulating DC-mediated Treg cell homeostasis and autoimmunity.

#### 5.2.4. Glucocorticoids

Glucocorticoids are a class of steroid hormones synthesized in the adrenal cortex that act in the regulation of glucose levels in the blood but have feedback properties that inhibit inflammation through modulation of gene transcription [125]. Glucocorticoids have been effectively used in the treatment of new onset T1D. Prednisone, combined with azathioprine, was shown to improve *β* cell function in new-onset T1D patients [126–128]. As glucocorticoids have been shown to downregulate dendritic cell function both* in vitro* and* in vivo*, they may continue to occupy a significant role in the suppression of tissue-specific autoimmunity [129].

### 5.3. Antibodies That Suppress Dendritic Cell Function

Targeted antibody immunotherapies hold great promise for the treatment and cure of tissue-specific autoimmune diseases [130]. Antibodies that bind DC costimulatory factors CD83, CD86, and CD80 were shown to arrest DC maturation by blocking DC costimulatory factor interaction with CD28 receptors on autoreactive T cells, thereby reducing or inhibiting DC stimulation of effector T cell development [131, 132]. Psoriasis, a form of skin autoimmunity, is characterized by DC induction of autoreactive Th1 and Th17 effector cell differentiation. Administration of antibodies specific for the p40 subunit of IL-12 and IL-23 (anti-IL-12p40) reduced mRNA expression of proinflammatory cytokines and chemokines in psoriatic skin lesions following a single administration of anti-IL-12p40. These studies demonstrate the efficacy of anti-IL-12 antibody immunotherapy for suppression of chronic inflammatory skin disorders [133]. Further studies have shown that anti-IL-17 antibodies are effective in suppression of experimental uveoretinitis and rheumatoid arthritis [134, 135]. In T1D, anti-IL-17 antibodies were shown to inhibit diabetes during the effector phase of disease progression in NOD mice (at 10 weeks of age), but not during the initiation of disease (in mice less than 5 weeks of age). This data suggests that DC stimulation of IL-17 secreting Th17 cells does not occur until T1D disease progression [136].

Anti-CD3-specific antibodies demonstrate a unique capacity to restore self-tolerance in established autoimmunity. They induce long-term remission of overt diabetes both in nonobese diabetic (NOD) mice and in human T1D [137]. The potency of anti-CD3-specific monoclonal antibody therapy in mice and humans results from its ability to reestablish immune homeostasis in treated individuals, likely through a concerted dendritic cell and regulatory T-cell-mediated mechanism [138]. Anti-CD3 binds the T-cell receptor- (TCR-) CD3 complex (also termed antigenic modulation) and induces apoptosis of activated autoreactive T cells. This T cell clearance allows for homeostatic remission, survival, and expansion of Treg cell populations which effectively control pathogenic effectors including DCs [138]. There is compelling evidence that regulatory T cells exert their control over pathogenic T cells through suppression of DC activation rather than from direct T cell-T cell interactions. The immunoregulatory cytokine TGF-*β*, which in this model is not only produced by regulatory T cells but also potentially by DCs and other stromal cell types, is an ideal candidate cytokine for the maintenance of a broad anti-inflammatory environment through its action on effector T cells, regulatory T cells, and DCs [138].

### 5.4. Dendritic Cell Gene Therapy

Suppression of tissue-specific autoimmunity may be accomplished based on an innovative therapeutic strategy in which susceptible subjects are treated with their own mDCs. Monocytes isolated from the patient can be differentiated* in vitro* to obtain large numbers of moDCs that can be transfected with genes encoding immunosuppressive cytokines such as IL-10, TGF-*β*, or IL-4. In addition to suppression of inflammatory cytokine synthesis, the activated DCs would provide an element of safety because they have a limited lifetime of approximately 5 days and therefore would have only a transient effect on the immune response [139]. Additional studies have demonstrated prevention and reversal of type 1 diabetes in NOD mice using costimulation impaired, immunosuppressive bone marrow-derived DCs generated* ex vivo* with a mixture of antisense oligonucleotides targeting the primary transcripts of DC costimulatory factors CD40, CD80, and CD86 [140]. Phase 1 clinical trials show that the vaccine is well tolerated in patients [141].

### 5.5. Immunosuppressive Vaccines

Parenteral vaccination is generally considered to be the most effective form of protection against infectious diseases. More recently, however, vaccination at mucosal surfaces and combinatorial vaccination strategies that link immunostimulatory molecules (adjuvants) to antigens have been developed to further enhance vaccine efficacy. Prominent among immunological enhancement strategies are the group of bacterial and plant AB toxins, which include shiga toxin, anthrax toxin, ricin toxin, the heat sensitive enterotoxin from* E. coli*, and the cholera toxin CTA and CTB subunits [142]. In contrast to the toxic CTA subunit, the nontoxic CTB subunit displays both carrier and mild immunostimulatory properties [143]. When linked to pathogen antigens, CTB can impart immunostimulatory properties that convey increased levels of immune system stimulation in response to the linked antigen. These vaccination strategies have been broadened further to include CTB linkage to “self” proteins, which paradoxically often result in enhanced immunological suppression of autoimmunity. Linkage of CTB to an autoantigen was shown to provide up to a 10,000-fold reduction in the amount of autoantigen required for generating immune-tolerance [142, 144, 145]. In T1D, for example, self-proteins become more strongly immunosuppressive when linked to CTB. In addition to its known capacity to induce a proinflammatory response, oral administration of CTB subunit coupled with insulin or GAD_35_ autoantigen was shown to induce immunological tolerance in NOD mice [142, 144, 146].

Morphological changes in DCs incubated with CTB coupled to an autoantigen included cell enlargement, elongation of dendrites, and increased migration of DCs to draining lymph nodes, as well as increased expression of dendritic cell B7-2/CD86 costimulatory molecules [142, 147]. In a recent finding in our laboratory, incubation of human immature moDCs with CTB-INS autoantigen fusion protein showed an increase in surface expression of TLR2 with no significant upregulation in TLR4 expression [148]. In contrast, inoculation of immature dendritic cells (iDCs) with CTB stimulated the biosynthesis of both CD86 and CD83 costimulatory factors demonstrating an immunostimulatory role for CTB in both DC activation and maturation. In comparison, incubation of iDCs with proinsulin partially suppressed DC activation, while incubation of iDCs with CTB-INS fusion protein suppressed iDC biosynthesis of both CD86 and CD83 costimulatory factors. Inoculation of iDCs with CTB-INS fusion protein was shown to dramatically increase secretion of the immunosuppressive cytokine IL-10 while suppressing synthesis of the proinflammatory cytokine IL12/23p40 subunit. This result suggests that linkage of CTB to proinsulin (INS) could play an important role in mediating DC guidance of Th0 cell development into Treg cells. Taken together, the experimental data suggests that TLR2 may play a dominant role in CTB-INS-mediated prevention of human T1D onset. Further, fusion of CTB to proinsulin was found to be essential for enhancement of immune suppression as codelivery of CTB and insulin did not significantly inhibit dendritic cell CD86 biosynthesis. Thus, the experimental data supports the hypothesis that CTB-autoantigen-mediated suppression of islet *β* cell inflammation and hyperglycemia development is dependent on CTB stimulation of dendritic cell TLR2 receptor activation and coprocessing of both CTB and the autoantigen in the same DC [148].

Our laboratory also found that linkage of CTB to a 5 kDa C-terminal protein fragment of the major diabetes autoantigen GAD_35_ can block DC functions including biosynthesis of costimulatory factor proteins CD86, CD83, CD80, and CD40 and secretion of the inflammatory cytokine IL-12 [149]. Inoculation of iDCs with CTB-GAD_35_ protein dramatically suppressed levels of CD86, CD83, CD80, and CD40 costimulatory factor protein biosynthesis in comparison with iDCs inoculated with GAD_35_ alone. Surprisingly, incubation of iDCs in the presence of the CTB-autoantigen and the strong immunostimulatory molecules PMA and Ionomycin revealed that CTB-GAD_35_ was capable of arresting PMA + Ionomycin induced DC maturation and activation. Consistent with this finding, CTB-GAD_35_-mediated suppression of DC maturation was accompanied by a dramatic decrease in the secretion of the proinflammatory cytokines IL-12/23p40 and IL-6 and a significant increase in secretion of the anti-inflammatory cytokine IL-10. Taken together, the experimental data suggests that linkage of the weak adjuvant CTB to the dominant type 1 diabetes autoantigens INS and GAD inhibits DC maturation through downregulation of major DC costimulatory factors and inflammatory cytokine biosynthesis. These experimental results also emphasize the possibility that CTB-autoantigen fusion proteins enhance DC priming of Th0 cell differentiation into Treg cells. The above described immunological phenomena establish a basis for improvement of adjuvant augmented multicomponent subunit vaccine strategies capable of complete suppression of organ-specific autoimmune diseases* in vivo* [149].

### 5.6. DC Suppression of Autoimmunity through Indoleamine 2,3 Dioxygenase (IDO)

The first enzyme in the tryptophan degradation pathway, indoleamine 2,3 dioxygenase, (IDO) may be an important contributor to DC-mediated suppression of autoimmunity. IDO is the rate-limiting catabolic enzyme encoded by the IDO1 gene responsible for the degradation of L-tryptophan (L-Trp) to N-formyl kynurenine and its further degradation products [150]. IDO was shown to inhibit DC maturation through tryptophan starvation via a generalized reduction in cellular energetics and through the generation of secreted kynurenines shown to be effective in stimulating T cell apoptosis and Treg proliferation [125–128]. Further, it was recently found that the tryptophan metabolite 3-hydroxyanthranilic acid (3-HAA) directly inhibits DC activation and is responsible for suppression of inflammatory Th1 cell functions [151]. Treatment with 3-HAA was shown to significantly reduce production of the proinflammatory cytokines IL-12, IL-6, and TNF-*α* in bone marrow-derived dendritic cells stimulated with LPS. The role of IDO in dendritic cell function may differ among DC subsets because the ability of DCs to produce IDO does not seem to be equally distributed among the various DC subsets. The CD8*α* positive DCs in mice were shown to express higher amounts of IDO in comparison with CD8*α*-negative DCs. In response to IFN*γ*, CD8*α*-positive DCs were shown to rapidly express IDO and establish immunological tolerance [152–154].

The immunosuppressive activity of IDO was first speculated to be solely a function of the physical depletion of tryptophan from the intracellular environment, thus starving DCs, T cells, and other effector cells of the immune system. Tryptophan starvation is sensed in eukaryotic cells through activation of the general control nonrepressed 2 (GCN2) kinase, which directly binds uncharged tRNAs [152, 155]. Tryptophan depletion was shown to result in the induction of the GCN2 pathway, the downregulation of CD3 zeta-chain in CD8^+^ T cells, and inhibition of Th17 cell differentiation [152, 156, 157]. An additional mechanism for IDO stimulated immune suppression resides in the inhibitory effect of kynurenines on T cell and natural killer cell proliferation [158, 159]. Equally important was the observation that kynurenines stimulate the upregulation of Treg cell proliferation that can further inhibit DC activation [160].* In vitro* cell culture experiments further demonstrate the immunosuppressive nature of IDO showing that elevated IDO activity can permit tumor cell escape from immune surveillance through depletion of L-Trp in the DC and T cell microenvironment [161].* In vivo* experiments have shown that IDO knockout mice experience acute rejection of transplanted MHC mismatched grafts, while wild-type mice with high tryptophan catabolism experienced long-term graft survival [162]. Further emphasizing the requirement for IDO in suppression of DC activation, experimental reduction in the levels of pDCs in the pancreas of NOD mice was shown to be accompanied by increased insulitis and a localized reduction in IDO levels [76]. Together, this data confirms the role of IDO as a strong immunosuppressive mediator in tissue-specific autoimmunity.

## 6. Concluding Remarks

Dendritic cells were first observed to be a novel class of antigen presenting cells by Ralph Steinman almost a half century ago. Since their discovery, however, considerable experimental data has accumulated describing their cytology and biological functions. Resulting from these studies, an increasing number of DC subtypes have been identified that provide a broad diversity in antigen presentation to lymphocytes of the adaptive arm of the immune system. The unique properties of DC presentation of antigens to naïve T cells and guidance of their differentiation into pro- or anti-inflammatory effector T cells have helped clarify the role of DCs as key mediators of protective immunity. Assessment of DC functions in the initiation and prevention of autoimmunity will continue to reveal elements that contribute to their role in maintenance of immunological homeostasis. The development of promising DC-based therapeutic strategies will lead to more effective and safer prevention and treatment for an increasing number of autoimmune disorders. Further analysis of mechanisms underlying DC activation and maturation will lead to a more complete understanding of how DCs function in the guidance of naïve T cell differentiation into proinflammatory or anti-inflammatory lymphocytes that exacerbate or inhibit autoimmunity. Finally, based on the present rate of accumulation of experimental data on dendritic cell cytology and functions, it is likely the number of DC subsets and our knowledge of their participation in the initiation and suppression of tissue-specific autoimmunity will continue to increase.
